# Vestibular status in partial deafness

**DOI:** 10.1016/j.bjorl.2019.09.012

**Published:** 2019-11-20

**Authors:** Magdalena Sosna, Grazyna Tacikowska, Katarzyna Pietrasik, Henryk Skarzynski, Piotr H. Skarzynski

**Affiliations:** a Institute of Physiology and Pathology of Hearing, Otorhinolaryngosurgery Clinic, Warsaw, Poland; b Institute of Physiology and Pathology of Hearing, Department of Otoneurology, Warsaw, Poland; c Institute of Physiology and Pathology of Hearing, World Hearing Center, Kajetany, Poland; d Institute of Sensory Organs, Kajetany, Poland; e Medical University of Warsaw, Heart Failure and Cardiac Rehabilitation Department, Warsaw, Poland

**Keywords:** Partial deafness, Cochlear implantation, Vestibular evoked myogenic potential, Video head impulse test

## Abstract

**Introduction:**

The hair cells of the cochlea and the vestibulum are closely connected and may be susceptible to the same noxious factors. The relationship between their function has been a continuing field of investigation. The indications for cochlear implantation have been broadened and now include the patients with partial deafness. This raises the question of their vestibular status.

**Objective:**

The aim of the study was to investigate whether there is any difference between the vestibular function of patients with low frequency residual hearing and those with totally deaf ears.

**Methods:**

A total of 360 ears with profound sensorineural hearing loss were analysed before cochlear implantation. The patients were divided into four groups, according to their low frequency residual hearing (Group 1 ‒ normal or slightly elevated low frequency residual hearing; Group 2 ‒ elevated threshold but still usable hearing at low frequencies; Group 3 – non-functional residual hearing; Group 4 ‒ no detectable hearing threshold within the limits of the audiometer). The patients underwent vestibular tests: cervical vestibular evoked myogenic potential, ocular vestibular evoked myogenic potential, caloric test and video-head impulse test.

**Results:**

The rates of elicited responses in cervical vestibular evoked myogenic potential were as follows: in Group 1 (59.3 %); Group 2 (57.5 %); Group 3 (35.2 %); Group 4 (7.7 %). For ocular vestibular evoked myogenic potential the percentage of correct outcomes was: Group 1 (70.8 %); Group 2 (56.0 %); Group 3 (40.0 %); Group 4 (14.3 %). For the caloric test we counted normal responses in 88.9 % of Group 1; 81.6 % of Group 2; 57.9 % of Group 3; 53.3 % of Group 4. For video-head impulse test we also found markedly better results in Group1, followed by Group 2, and much worse in Group 3 and 4.

**Conclusion:**

Patients with partial deafness not only have a better cochlea but also better vestibular function, which needs to be protected. In summary, the better the low frequency residual hearing, the better the vestibular status.

## Introduction

The hair cells of the cochlea and the vestibular organ are closely connected phylogenetically and anatomically, as they are both located in the membranous labyrinth and filled with the same inner ear fluid. Indeed, the relationship between cochlear and vestibular function has been a field of investigation for many years.^1^, ^2^, ^3^, ^4^, ^5^, ^6^, ^7^, ^8^ The cochlea and the vestibule can both be susceptible to noxious factors like infectious agents, ototoxic drugs, trauma, or insufficiency in inner ear blood supply. The coexistence of hearing loss and vestibular dysfunction has been well described in the literature.^1^, ^2^, ^3^, ^4^, ^5^, ^6^, ^7^, ^8^ Many genetic mutations cause hearing loss with coexisting vestibular impairment: COCH gene mutation, PAX3, MITF, SOX10 (Waardenburg syndrome), MYO7A, USH1C, CDH23, PCDH15, USH1G, USH2A, and USH3A (Usher syndrome I, III). Partial or total vestibular damage is also described in GJB2 mutations,^3^, ^4^ congenital rubella,^5^ bacterial meningitis.^6^

The battery of otoneurological tests, that are currently in clinical use enables us to assess each part of vestibulum separately:^9^ sacculus with cVEMP (cervical Vestibular Evoked Myogenic Potential) that measures vestibulo-collic reflex,^10^ utricle – with oVEMP (ocular Vestibular Evoked Potential) which measures vestibulo-ocular reflex,^11^ lateral semicircular canal – with caloric test for low-frequency stimulus and with rotatory chair or vHIT (video Head Impulse Test) for high-frequency stimulus as well as superior and posterior semicircular canal with Vhit.^12^

According to previous reports based on caloric tests and rotatory chairs, the overall incidence of vestibular deficits in patients with profound hearing loss ranges from 20 % to 40 %.^5^, ^7^ More recent research with an extended battery of otoneurological tests (including VEMPs) found that this percentage could be as high as 85 %.^3^, ^4^, ^6^, ^8^

Indications for cochlear implantation have steadily been broadened, and now include patients with low-frequency residual hearing as good candidates. This raises the question of their vestibular status. The aim of this study was to investigate whether there was any difference between the vestibular function of patients with low frequency residual hearing and those with totally deaf ears and whether there was any association between the degree of low-frequency hearing loss and vestibular function.

## Methods

Exactly 225 patients with profound sensorineural hearing loss in at least one ear (with or without low frequency residual hearing) were enrolled in the study. Of them, 135 had bilateral profound hearing loss, so a total of 360 ears were analysed.

The patients underwent vestibular tests: cVEMP, oVEMP, caloric test, and vHIT, which were part of an evaluation for cochlear implantation. The study was approved by the institutional board’s ethics committee KB/15/2014. At the beginning of our study, in 2014–2015, only cVEMPs and caloric test assessment were performed; after 2015 we included oVEMP and vHIT testing as well. All the patients gave written informed consent. After cochlear implantation the vestibular status of the participants was checked again with the same test battery according to the following schema: cVEMP, oVEMP 1–3 months, vHIT, caloric test 4–6 months postoperatively. We measured also the hearing preservation 6 months after the cochlear implantation using the following formula^13^ HP = [1-(PTApost-PTApre)/(PTAmax-PTApre))*100 %, where PTApre is the pure tone average measured preoperatively, PTApost is the pure tone average measured postoperatively, and PTAmax is the maximum level generated by a standard audiometer. The HP (hearing preservation) values were divided into: loss of hearing (no detectable hearing), minimal HP (range, 1 %–25 %), partial HP (26 %–75 %), and complete HP (> 75 %).^13^

However, for the purposes of this paper, the detailed analysis was restricted to the preoperative results of vestibular tests and their association with the preoperative hearing.

**Figure 1:**
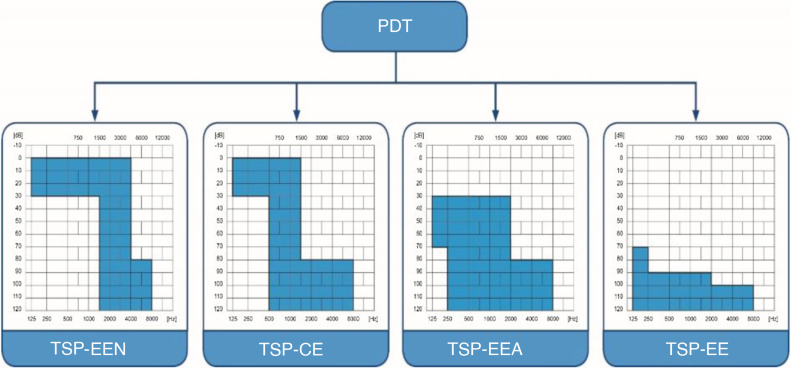
Classification of partial deafness treatment. PDT-ENS, Partial Deafness Treatment – Electro-Natural Stimulation; PDT-EC, Partial Deafness Treatment – Electrical Complement; PDT-EAS, Partial Deafness Treatment – Electro-Acoustic Stimulation; PDT-ES, Partial Deafness Treatment – Electrical Stimulation.

**Figure 2:**
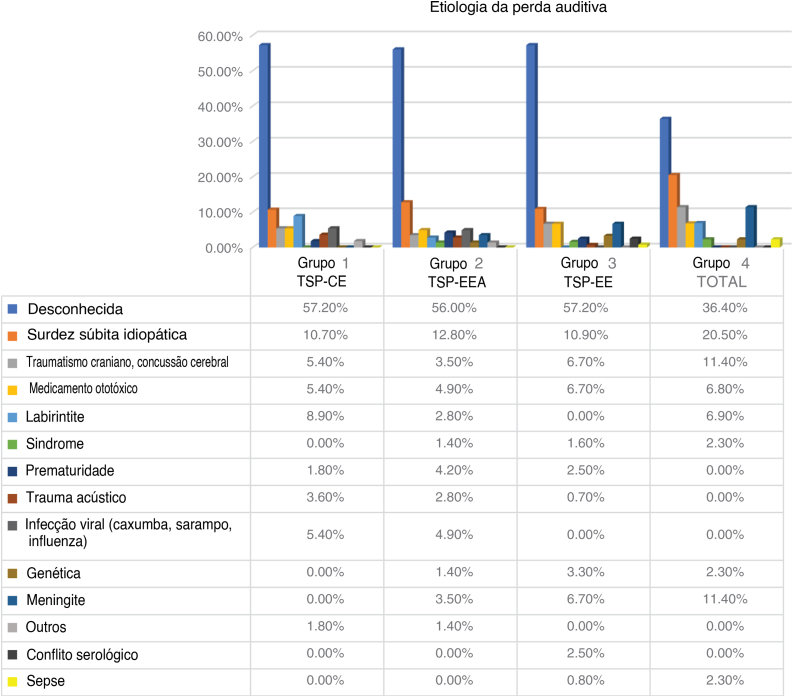
Etiology of hearing loss.

The patients were divided into four groups according to their hearing thresholds and their low frequency hearing. We based the division on the Partial Deafness Treatment (PDT) classification (Figure 1, Figure 2).^14^, ^15^, ^16^, ^17^ The classification refers the patients who are partially deaf, so have detectable hearing thresholds within the limits of audiometer that need to be protected and result in different surgical strategies of cochlear implantation according to the degree of preserved hearing. Electro-Natural Stimulation (PDT-ENS) refers to patients with normal or only slightly elevated thresholds in low and mid frequency bands, who need electrical complementation with a very short electrode;^15^, ^16^ Electrical Complement (PDT-EC) ‒ patients with normal or only slightly elevated thresholds at low frequencies, who need electrical complementation with short electrodes and no amplification at apical region;^17^ Electro-Acoustic Stimulation (PDT-EAS) ‒ patients with low- and mid- frequency residual hearing who need amplification from a hearing-aid for low frequencies and electric stimulation from implanted electrode for mid and high frequencies^18^ and Electrical Stimulation (PDT-ES) – includes the patients with non-functional residual hearing who will profit only from electrical stimulation after insertion of long electrode.

In our study Group 1 fulfilled the audiological criteria for the PDT-EC group, Group 2 for PDT-EAS and Group 3 for PDT-ES, while Group 4 comprised patients with no detectable hearing thresholds within the limits of the audiometer. The pure tone averages for 125, 250, 500 and 1000 Hz (PTA LOW FREQ) and descriptive statistics of each group are set out in Table 1.

**Table 1:** Descriptive statistics of all the groups.

	Number (Percentage %)	PTA LOW FREQ (dbHL)	Age range	Average age (Std. Deviation)	Sex (Female:Male ratio)
Group 1 PDT-EC	56 (15.6 %)	27.61 (±11.79)	9‒74	47.31 (±15.48)	30:26
Group 2 PDT-EAS	141 (39.2 %)	66.76 (±13.63)	9‒80	45.24 (±20.13)	71:70
Group 3 PDT-ES	119 (33.1 %)	93.93 (±7.80)	9‒84	42.52 (±18.82)	55:64
Group 4 Total	44 (12.2 %)	‒	14‒77	45.54 (±16.94)	21:23

PTA LOW FREQ, Average Pure Tone Audiometry for 125 Hz, 250 Hz, 500 Hz, 1000 Hz.

The exclusion criteria were: an already implanted ear, middle ear pathology such as a history of otosclerosis, ossicle damage or immobilization, status of post canal wall down tympanoplasty, any air-bone gap in pure tone audiometry, perforation of the ear drum, ‘third window syndrome’ (superior canal dehiscence syndrome), large aqueductus vestibuli, known retrocochlear pathology, or neurological disease that is known to affect VEMP responses (cerebellum pathology, demyelinating disease, neurodegenerative disease), neck muscles stiffness.

### cVEMPs

**Figure 3:**
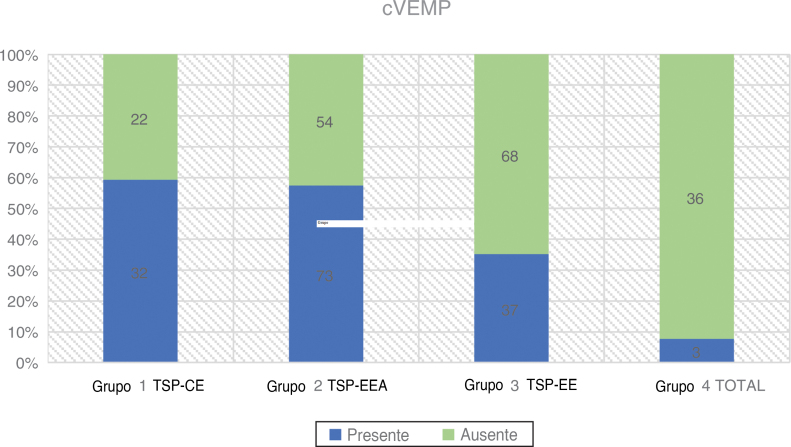
cVEMPs in partial deafness.

Stimulation was provided monaurally through insert tips with a 500 Hz tone burst at 97 dBnHL and stimulation rate of 5.1 Hz, stimulation gate 2:2:2. A set of 200 stimuli were averaged. The patient was seated and asked to rotate his head 45° away from the stimulated ear in order to achieve constant tonic contraction of the Sternocleidomastoideus (SCM) muscle. An SCM contraction level of 50–150 μV was maintained during the whole examination using visual biofeedback derived from the software. The two active electrodes were placed at the midpoint between the termination of the muscle at the mastoid and its origin at the sternum, the inverting electrode was placed between the sternoclavicular joints, and the ground electrode was attached to the forehead. The impedance of the electrodes was maintained below 2.5 kΩ. The response was regarded as present if two repeatable electromyographic patterns were elicited with two recognizable waveforms P1 and N1 (Fig. 3 and Table 2).

**Table 2:** cVEMPs in partial deafness.

	Number of patients	cVEMP		Age range	Average age (Std. Deviation)	PTA LOW FREQ dBHL (Std. Deviation)
		Present	No response			
Group 1 PDT-EC	54	32 (59.3 %)	22 (40.7 %)	9‒74	47.44 (±15.76)	28.19 (±11.60)
Group 2 PDT-EAS	127	73 (57.5 %)	54 (42.5 %)	9‒75	43.59 (±19.82)	67.09 (±13.65)
Group 3 PDT-ES	105	37 (35.2 %)	68 (64.8 %)	9‒84	42.70 (±18.56)	93.98 (±7.71)
Group 4 TOTAL	39	3 (7.7 %)	36 (92.3 %)	14‒70	44.61 (±15.85)	‒

PTA LOW FREQ, Average Pure Tone Audiometry for 125 Hz, 250 Hz, 500 Hz, 1000 Hz.

### oVEMPs

**Figure 4:**
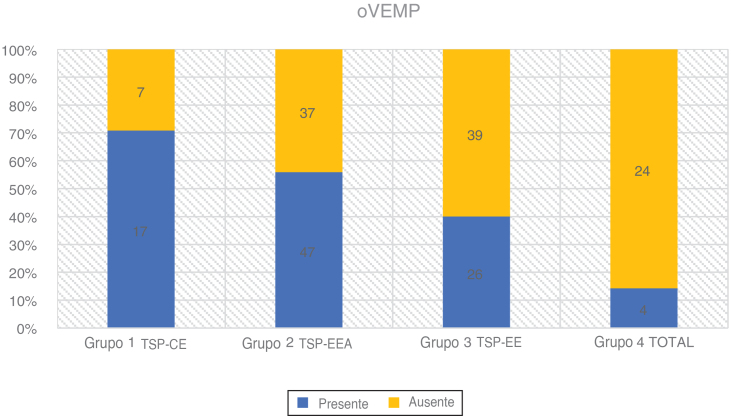
oVEMPs in partial deafness.

oVEMPs were measured using a 500 Hz tone burst at 97 dBnHL, 2:2:2 stimulation gate, stimulation rate of 5.1 s, signal averaging of 500 stimuli. The active electrodes were placed infraorbitally in the midline of the eye, the reference electrode was on the chin, and the ground electrode was on the forehead. The response was recorded contralaterally and the impedance of the electrode was maintained below 2.5 kΩ. The patient was seated and asked to gaze 35° vertically during the recording. The response was regarded as present if two repeatable patterns were recorded with two recognizable waveforms N1 and P1 (Fig. 4 and Table 3).

**Table 3:** oVEMPs in partial deafness.

	Number of patients	oVEMP		Age range	Average age (Std. Deviation)	PTA LOW FREQ dBHL (Std. Deviation)
		Present	No response			
Group 1 PDT-EC	24	17 (70.80 %)	7 (29.20 %)	9‒68	44.62 (±16.70)	23.85 (±9.42)
Group 2 PDT-EAS	84	47 (56.00 %)	37 (44.00 %)	9‒74	46.17 (±18.20)	67.08 (±14.32)
Group 3 PDT-ES	65	26 (40.00 %)	39 (60.00 %)	16‒84	44.43 (±18.63)	94.83 (±7.11)
Group 4 TOTAL	28	4 (14.30 %)	24 (85.70 %)	16‒70	46.37 (±17.40)	‒

PTA LOW FREQ, Average Pure Tone Audiometry for 125 Hz, 250 Hz, 500 Hz, 1000 Hz.

### Caloric test

**Figure 5:**
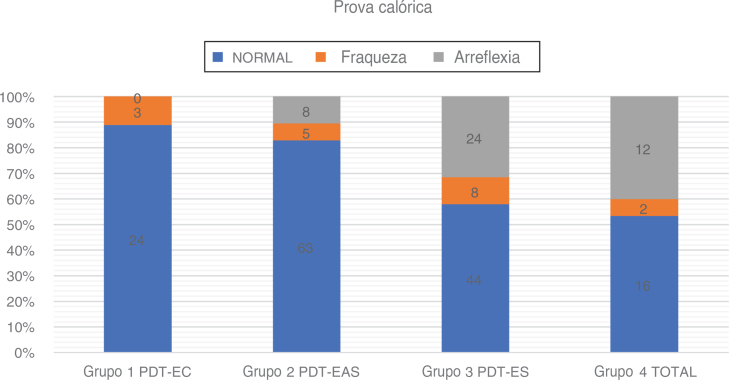
Caloric test in partial deafness. Black-white version.

We used Fitzgerald–Hallpike bithermal caloric stimulation, with two stimulations (44 °C and 30 °C) for 30 s. The second test was performed after a rest of at least 8 min from the first. The patients were supine with 30° elevation of the upper body. Unilateral Weakness (UW) and Slow Component Velocity (SCV) were recorded on both sides. The degree of canal paresis (UW) was calculated based on Jongkees’ formula, with 75 % > UW > 25 % judged as a weakened response and UW > 75 % as areflexia. In case of weak caloric responses bilaterally, areflexia was claimed if total caloric response (the sum of SCV for warm and cold water) <12°. In case of doubt, we performed additionally rotatory chair test or relied on vHIT in plane of lateral semicircular canals to distinguish between physiologically weak caloric responses and areflexia (Fig. 5 and Table 4).

**Table 4:** Caloric tests results in partial deafness.

	Number	Caloric tests			Age range	Average age (Std. deviation)	PTA LOW FREQ dBHL (Std. Deviation)
		Normal	Weakness	Areflexia			
Group 1 PDT-EC	27	24 (88.90 %)	3 (11.10 %)	0 (0.00 %)	31‒74	52.16 (±13.21)	23.05 (±7.52)
Group 2 PDT-EAS	76	63 (82.90 %)	5 (6.60 %)	8 (10.50 %)	12‒80	49.00 (±18.95)	64.14 (±14.43)
Group 3 PDT-ES	76	44 (57.90 %)	8 (10.50 %)	24 (31.60 %)	10‒75	44.42 (±17.83)	94.72 (±7.30)
Group 4 Total	30	16 (53.30 %)	2 (6.70 %)	12 (40,00 %)	16‒77	47.06 (±18.10)	‒

PTA LOW FREQ, Average Pure Tone Audiometry for 125 Hz, 250 Hz, 500 Hz, 1000 Hz.

### vHIT

The patient was seated and asked to keep staring at a spot. Then an abrupt, unpredictable, small angle (about 10°–20°) head movement was given in three planes: horizontal, Left Anterior–Right Posterior (LARP) and Right Anterior–Left Posterior (RALP). In every case 20 impulses were delivered with a minimal peak head velocity of 150°/s. Normal gain (the quotient of head movement speed and eye movement speed) ranged from 0.6 to 1.2. A gain below 0.6 or the presence of a covert or overt saccade was treated as evidence of damage to the particular semicircular canal (Table 5).

**Table 5:** vHIT results. Number (Percentage %).

	Lateral			Posterior			Anterior			Age range	Mean age (Std. Deviation)	PTA LOW FREQ dBHL (Std deviation)
	Damage	Normal	χ^2^	Damage	Normal	χ^2^	Damage	Normal	χ^2^			
Group 1 PDT-EC	0 (0.0)	24 (100)	20.21; p < 0.001	4 (16.7)	20 (83.3)	842; p < 0.05	2 (8.3)	22 (91.7)	884; p < 0.05	30‒74	51.95 (±13.56)	25.00 (±7.45)
Group 2 PDT-EAS	4 (6.8)	55 (93.2)		9 (15.5)	49 (84.5)		5 (8.8)	52 (91.2)		10‒75	47.13 (±19.90)	64.70 (±12.93)
Group 3 PDT-ES	15 (30.6)	34 (69.4)		15 (31.3)	33 (68.8)		11 (22.9)	37 (77.1)		10‒75	41.81 (±18.33)	93.68 (±7.54)
Group 4 Total	7 (35.0)	13 (65.0)		8 (44.4)	10 (55.6)		6 (33.3)	12 (66.7)		18‒70	45.02 (±17.69)	--------

PTA LOW FREQ, Average Pure tone Audiometry for 125 Hz, 250 Hz, 500 Hz, 1000 Hz.

### Statistical analysis

The Chi-Square test for independence was conducted to determine whether there is a significant association between affiliation to a particular hearing group and the results of vestibular tests and logistic regression to describe the association between low-frequency residual hearing (measured as PTA for 125 Hz, 250 Hz, 500 Hz, 1000 Hz; PTA LOW FREQ) and vestibular function.

As the results of vestibular testing depend on age, especially for VEMP tests,^19^, ^20^, ^21^, ^22^ we used additionally the Kruskal-Wallis test to compare all four groups in terms of age. For statistical analysis, IBM SPSS Statistics v. 24 software was used and *p* < 0.05 was established as statistically significant.

## Results

Among 360 ears of 225 patients enrolled in the study, 325 ears were assessed with cVEMP, 201 with oVEMP, 209 with caloric test and 152 with vHIT. The discrepancies between the number of examined ears with each test were caused by: inability to follow properly the protocol of the particular exam which made its result not reliable (to keep the proper tension of SCM muscle for cVEMP, to keep the eye fixated on one point during head movements or to achieve the proper velocity of head movements in vHIT), disagreement of the patients for the whole battery of test (especially for caloric tests, as the most vertigo-provoking), intolerance of the tests (for example caloric stimulation), introducing oVEMP and vHIT later into the protocol. The summary of examined ears before and after the cochlear implantation is depicted in Table 6. Among n = 142 ears that had any detectable hearing before cochlear implantation (Group 1, 2 and 3) and were analyzed after the cochlear implantation: n = 60 (42.25 %) preserve completely hearing, n = 73 (51.40 %) ‒ partially, n = 6 (4.22 %) ‒ minimally and n = 3 (2.11 %) had no detectable hearing in 6 months follow-up.

**Table 6:** Number of examined ears for each vestibular test.

	cVEMP	oVEMP	Caloric test	vHIT Lateral	vHIT Posterior	vHIT Anterior
Number of ears examined before cochlear implantation	325	201	209	152	148	147
Number of ears with absent response before cochlear implantation	180 (55.38 %)	107 (53.23 %)	44^a^ (21.05%)	26 (17.10 %)	36 (23.32 %)	24 (16.33 %)
Number of ears with present response before cochlear implantation	145 (44.61 %)	94 (46.77 %)	165^b^ (78.95%)	126 (82.89 %)	112 (75.67 %)	123 (83.67 %)
Number of ears examined after cochlear implantation that had the response preoperatively^c^	72	47	37	27	23	25
Number of ears examined after cochlear implantation with maintained responses^c^	58 (80.56 %)	37 (78.72 %)	32 (86.49 %)	25 (92.60 %)	21 (91.30 %)	24 (96.00 %)
Number of ears examined after cochlear implantation with lost responses^c^	14 (19.40 %)	10 (21.30 %)	5^d^ (13.5%)	2 (7.40 %)	2 (8.70 %)	1 (4.00 %)

a Only areflexia in caloric response.

b Both normal and weakened caloric response.

c Not analyzed further in this paper.

d Change in unilateral weakness >25 % towards the implanted ear.

### cVEMPs

A total number of 325 ears were assessed preoperatively. The highest rate of positive responses was reported in Group 1 (59.3 %), followed by Group 2 (57.5 %). In Group 3 only 35.2 % of patients had present responses, while in Group 4 the percentage of registered VEMPs decreased to 7.7 %. The relationship between affiliation to a group and the rate of elicited cVEMP responses was statistically significant using a Chi-Square test (χ^2^ = 38.45; *p* < 0.001).

The average age in all four groups was similar, the highest in Group 1, where we had the highest rate of the correct cVEMPs responses. The difference in mean age between the groups was not statistically significant: χ^2^ = 2.21; *p* = 0.530 (*p* > 0.05). Logistic regression showed that the incidence of cVEMP responses was dependent on age and PTA LOW FREQ (*p* < 0.001 for both of them). Every 10 dBHL less in hearing thresholds measured as PTA LOW FREQ increased the odds of having cVEMP response 1.27 times. (OR = 1.27)

### oVEMPs

Again, the rate of positive VEMP responses decreased proportionately with the deterioration of low-frequency residual hearing in 201 ears we examined. We observed the highest rate of registered responses in Group 1 (70.8 %) and Group 2 (56.0 %). Group 3 had 40.0 % detected responses and Group 4 only had 14.3 %. According to a Chi-square test, the grade of low-frequency residual hearing had a significant effect on the rate of elicited responses (χ^2^ = 21.49; *p* < 0.001).

The Kruskal-Wallis test showed that the difference in age between the groups was statistically insignificant: χ^2^ = 0.53; *p* = 0.913 (*p* > 0.05). The rate of elicited oVEMPs depended on age (*p* < 0.001) and PTA for low frequency residual hearing (*p* < 0.001). Every drop by 10 dBHL in PTA LOW FREQ was associated with the odds of eliciting oVEMP being 1.22 times higher (OR = 1.22).

### Caloric tests

A caloric test was conducted on 209 ears. A relationship between the degree of low-frequency residual hearing and function of the lateral semicircular canal was also apparent. The rate of normal caloric responses was highest in Group 1 (88.9 %) and in Group 2 (81.6 %). Lateral semicircular canal function was weakest in Group 3 (57.9 %) and in Group 4 (53.3 %). Statistical analysis with a Chi-Square test showed that the low-frequency residual hearing had the impact on the rate of normal caloric responses: χ^2^ = 25.75; *p* < 0.001

Among all patients who underwent a caloric test, the age difference between the groups was statistically insignificant: χ^2^ = 4.39; *p* = 0.222 (*p* > 0.05). Logistic regression proved the statistically significant correlation between the result of caloric test and low frequency residual hearing (*p* = 0.0001), while the age was on the border of statistical significance (*p* = 0.050). Decrease by 10 dB in PTA LOW FREQ implicated the odds of having normal caloric response rather than weakened or areflexia being 1.46 times higher (OR = 1.46).

### vHIT

vHIT results were markedly the best in Group 1, followed by Group 2 and much worse in Group 3 and 4. We checked the vestibulo-ocular reflex with vHIT in 152, 148, 147 ears in the plane of lateral, anterior and posterior semicircular canal respectively. In PDT-EC group the semicircular canal function was fully preserved in 83.3 %. In PDT-EAS the percentage of cases with preserved all semicircular canals function was not less than 84.5 %. In PDT-ES the rate of correct vHIT results in all planes was 68.8 %. The group Total counted at least 55.6 % normal responses for all canals. The differences between the groups were statistically significant both for anterior, posterior and lateral semicircular canal.

In the case of lateral, anterior and posterior semicircular canal, the incidence of correct results of vHIT were not associated with age (correspondingly *p* =  0.760, *p* = 0.477, *p* =  0.674), but dependent on the average of low-frequency hearing threshold (correspondingly *p* <  0.001, *p* =  0.004, *p* =  0.001). The odds of having maintained the vestibulo-ocular reflex in the plane of lateral, posterior and anterior semicircular canal increased respectively 1.71 (OR = 1.71), 1.24 (OR = 1.24) and 1.41 (OR = 1.41) times with every 10 dBHL less in PTA LOW FREQ.

## Discussion

Partial Deafness (PD) is a condition where hearing loss occurs in at least one frequency critical to speech understanding.^14^ For a number of years, it has been known that patients with partial deafness can profit from a Cochlear Implant (CI). At the very beginning only those with non-functional residual hearing at low frequencies were implanted with a CI. Then, with progress in technology, electrophysiological knowledge and surgical techniques (hearing preservation techniques: the round window approach, soft electrodes, micropuncture of the round window, perioperative steroids administration),^14^, ^18^, ^23^, ^24^, ^25^ it became possible for patients with usable hearing at low frequencies to receive both electrical stimulation by a CI and acoustical amplification of their residual hearing (Partial Deafness Treatment–Electro-Acoustic Stimulation; PDT-EAS). After further years of improving surgical techniques and gaining more experience, the patients with normal or only slightly elevated hearing at low-frequencies but who need an electrical complement – a CI having shorter electrodes for mid- and high-frequencies were also included and classified as Partial Deafness–Electric Complement.^17^ Recently, a few cases of CI recipients who had normal hearing up to 1500 Hz have been described and classified as Partial Deafness Treatment–Electro-Natural Stimulation (PDT-ENS).^15^, ^16^

Cochlear implantation in partial deafness raises the problem of preservation of residual hearing. In the present analysis, it is clear that this group of patients not only have a better cochlea but also good vestibular function that needs to be protected. It is true that the consequences of vestibular loss are not as serious as those of hearing loss. In cases of vestibular damage, compensation mechanisms in the central nervous system enable largely normal postural control and make the vast majority of vestibular postoperative symptoms only transient. However, as we recruit more elderly people for CI treatment, or those with preexisting vestibular or neurological disorders, the problem gains in importance, especially with bilateral implantation. The literature that touch on the issue of vestibular preservation after cochlear implantation reported loss of cVEMP responses in 0 %–76.47 %^26^, ^27^, ^28^, ^29^, ^30^, ^31^ and significantly reduced caloric responses in 0 %–72.40 %,^26^, ^27^, ^28^, ^29^, ^30^, ^31^, ^32^ even when hearing preservation techniques were adopted. That is why special care and counselling are recommended when qualifying a patient for cochlear implantation.

Our outcomes cannot be a direct indicator of vestibular status in deaf ears but show the statistical tendency. It is worth noting that at least 53.3 % patients without detectable residual hearing had normal later semicircular canal function which may worsen after cochlear implantation. They may be potentially treated as totally damaged ears where nothing more can be lost after the operation, while more than half of them still have relevant vestibular function. Our results point out that the otolith organs are more affected in profoundly deaf ears (sacculus in 55.38 % and utricle in 53.23 %) than the semicircular canals (between 16.33 %–23.32 % lack of response according to caloric test or vHIT). This may be clarified by anatomical proximity to the cochlea that makes the sacculus and utricle more susceptible to noxious factors.

There are very few comparable data in the literature. To our knowledge no other research has used such a range of vestibular tests to examine so many partially deaf patients.

Tribukait et al.^1^ showed that if hearing was better than 90 dB (PTA of 0.5, 1.0, and 2.0 kHz), vestibular function was often normal but for hearing levels of 100/120 dB, otolith function declined significantly. Hearing levels correlated more closely with otolith function, especially that of the utricle, than with lateral semicircular canal function.^1^ Lin and Young^33^ divided the patients with non-inherited and non-syndromic congenital sensorineural hearing loss into two groups according to the grade of hearing loss: > 90 dB (PTA, for 500 Hz, 1000 Hz, 2000 Hz, 3000 Hz) and ≤ 90 dB (PTA for 500 Hz, 1000 Hz, 2000 Hz, 3000 Hz). They discovered that the worse hearing group presented higher incidence of absent responses than the latter: cVEMP (80 % and 68 %), oVEMP (70 % vs. 20 %) and caloric tests (40 % vs. 5 %) respectively. Hearing loss with PTA ≥ 65 dB was conjoined with significantly worse vestibular function.^33^

Sandberg and Terkildsen^7^ found a correlation between the severity of hearing loss and the results of caloric tests (80 % normal caloric tests when PTA for 0.5, 1 and 2 kHz was < 90 dB but only 20 % normal caloric tests when PTA > 98 dB).

Limitations of our study stem from the fact that the VEMP test was conducted with air-conducted stimuli. To eliminate false negatives, we excluded all patients with coexisting conductive hearing loss. We have not introduced any age limitations. Some of our patients could have had absent or weak vestibular responses due to aging of the vestibular organ, especially in case of VEMPs. The average age was similar in each group and could not have affected the final result. The particular etiology of deafness in each group might have had some impact on the status of their vestibular function. In all our groups, the etiology of hearing loss was mostly ‘unknown’, but there seemed to be no strong preponderance in any group of a particular cause of hearing loss.

## Conclusion

Patients with partial deafness have better vestibular status than those with no detectable low-frequency residual hearing. In brief, we have found that the better the low frequency residual hearing, the better the vestibular status. If no strict audiological or anatomical criteria are present, then the preoperative vestibular status seems to be a good indicator of which side to operate.

## Funding

The research did not receive any specific grant from funding agencies in the public or commercial sectors.

## Conflicts of interest

The authors declare no conflicts of interest.
